# Inhibition of PI3K or AKT Differently Regulates Mesodermal Differentiation in P19CL6 Cells

**DOI:** 10.3390/biology15201887

**Published:** 2026-10-19

**Authors:** Siying Jiang, Yesui Li, Mingkuo Yang, Jinyu Wang, Ziyi Chen, Shuobing Zhang, Yanming Li, Tao Li

**Affiliations:** 1 School of Medical Technology and Translational Medicine, Hunan Normal University, Changsha 410013, China; 18874154382@163.com (S.J.); ysl971013@gmail.com (Y.L.);; 2 Department of Cardiology, Huaihe Hospital of Henan University, Kaifeng 475000, China; 3 Key Laboratory of Model Animals and Stem Cell Biology in Hunan Province, Hunan Normal University, Changsha 410013, China

**Keywords:** stem cells, cardiac differentiation, PI3K, AKT, Wnt

## Abstract

The directional differentiation of stem cells into cardiomyocytes displays distinct stage-specific characteristics. Multiple signaling pathways exhibit differential effects at various developmental stages in the regulation of cardiac differentiation. This study investigated the role and underlying mechanisms of the PI3K-AKT signaling pathway during both early and late stages of myocardial differentiation. Our results demonstrated that PI3K inhibitors promote early myocardial differentiation and enhance the expression of the mesodermal differentiation marker gene *Brachyury T* in P19CL6 teratoma cells. In contrast, AKT inhibitors exert the opposite effects. AKT activity is required for Wnt activation-induced *Brachyury T* expression, and Wnt inhibition also decreased AKT phosphorylation. Notably, both PI3K and AKT inhibitors suppress the late-stage myocardial differentiation. Mechanistically, PI3K inhibition promotes mesodermal differentiation in association with enhanced phosphorylation of GSK3β and activation of β-catenin. AKT kinase activity is indispensable for GSK3β phosphorylation and β-catenin activation. Furthermore, the subcellular localization of the PI3K p85 regulatory subunit undergoes significant changes before and after differentiation. Transcriptome sequencing revealed that treatment with PI3K inhibitors regulates multiple signaling pathways, including Wnt, Notch, and Nodal. Wnt3a expression is significantly upregulated upon PI3K inhibition. PI3K inhibitors did not affect the expression of pluripotency-related genes such as *Oct4* and *Sox2* but significantly suppressed the expression of growth factor receptor adaptor proteins such as *Grb7*. These findings suggest that PI3K plays a pivotal role in maintaining stem cell self-renewal and that inhibition of the PI3K pathway can activate Wnt, Notch, and Nodal signaling to promote mesodermal differentiation, thereby favoring the initiation of cardiac differentiation. AKT activation during mesoderm differentiation may occur independently of the PI3K pathway.

## Simple Summary

Inhibition of the PI3K pathway promotes mesodermal differentiation, whereas inhibition of the AKT pathway does the opposite. Mechanistic studies demonstrate that inhibition of the PI3K pathway activates the Wnt/β-catenin signaling pathway. Inhibition of the PI3K pathway can promote the expression of genes such as Wnt3a, Nodal, and Notch1, thereby facilitating mesodermal differentiation.

## 1. Introduction

Adult mammalian cardiomyocytes are terminally differentiated cells with negligible proliferative capacity, rendering them incapable of effectively replenishing lost myocardium following injury. Stem cell-based therapy has therefore garnered significant attention as a potential regenerative strategy for restoring damaged cardiac tissue, particularly in the context of myocardial infarction and heart failure.

The directed differentiation of pluripotent stem cells toward the cardiomyocyte lineage recapitulates key stages of embryonic cardiogenesis. Cardiac differentiation in vitro represents a continuous yet stage-specific developmental process governed by stringent intrinsic programming mechanisms and exogenous signal networks [1,2]. The process begins with the induction of mesoderm, marked by the expression of Brachyury T (T), followed by the specification of cardiac mesoderm. Subsequent activation of core cardiac transcription factors—including GATA4, NKX2-5, MEF2C, and TBX5—drives the commitment of cardiac progenitor cells, which ultimately differentiate into functional, spontaneously contracting cardiomyocytes [3].

The in vitro induction of myocardial differentiation from embryonic stem (ES) cells or induced pluripotent stem (iPS) cells is generally divided into two stages [2,4]. In the early stage, cells are cultured in suspension or as hanging droplets, during which pluripotent stem cells aggregate into clusters known as embryoid bodies (EBs). Within these EBs, undifferentiated cells progressively commit to mesoderm and subsequently to cardiac mesoderm. In the late stage, EBs are subjected to adhesion culture, allowing cells to migrate from the inner layers. Cardiac progenitor cells, which express specific myocardial differentiation transcription factors, continue to mature until they give rise to spontaneously beating cardiomyocytes.

A comprehensive “signaling pathway code” that governs cellular fate decisions and gives rise to diverse cell types, including smooth muscle cells, endothelial cells, and cardiomyocytes. During the multi-stage program of cardiomyogenesis, numerous signal transduction pathways are sequentially engaged, including Wnt, Notch, BMP, FGF, and Activin/Nodal signaling cascades [5]. At distinct developmental stages, exogenous signals exhibit the developmental stage-specific effects and dose-dependent responses. Multiple signaling pathways exert distinctly different biological effects during both the early and late stages of myocardial differentiation. The canonical Wnt signaling pathway promotes mesodermal differentiation during the early phase of cardiac differentiation; conversely, the early inhibition of Wnt signaling directs cells toward neural ectodermal lineage specification. In contrast, activation of the canonical Wnt signaling pathway during later stages suppresses myocardial differentiation and biases mesodermal cells toward hematopoietic lineage development [6]. A hallmark of the canonical Wnt signaling pathway is the cytoplasmic stabilization and nuclear translocation of β-catenin. In the absence of Wnt ligands, β-catenin is sequestered in a cytoplasmic complex with APC and Axin, where it undergoes phosphorylation by glycogen synthase kinase 3β (GSK3β). This phosphorylation event subsequently recruits an E3 ubiquitin ligase, leading to the ubiquitination and proteasomal degradation of β-catenin. Conversely, upon Wnt ligand binding to its cognate receptor complex, the degradation of β-catenin is effectively relieved. This results in the accumulation of β-catenin in the cytoplasm, followed by its translocation into the nucleus, where it partners with transcription factors of the TCF/LEF1 family to regulate target gene expression. Furthermore, β-catenin assembles with classical cadherins (such as E-cadherin) and other proteins, including α-catenin and p120-catenin, to form the adherens junction complex, which mediates cell–cell adhesion.

The Notch pathway likewise exhibits a biphasic effect, promoting myocardial differentiation in the early stage while inhibiting it in the later stage [7]. During ES cell-derived myocardial induction, Notch1, Notch4, and Jagged2 are stably expressed in early phases but undergo a rapid decline after the cells transition into the gastrula mesodermal stage [8]. Furthermore, the TGFβ/BMP family effector protein Smad2 also plays a dual-phase regulatory role throughout myocardial differentiation. At the early stage, Smad2 mediates the Nodal/Activin signaling pathway to promote mesodermal and endodermal differentiation [9,10]; however, during the later stage, it activates the TGFβ signaling pathway, thereby suppressing the expression of cardiomyocyte-specific genes [9,10]. Therefore, comprehensive elucidation of the signaling pathways in the stepwise cardiac induction will favor the improvement of cardiomyocyte differentiation efficiency or the specification of defined cardiac progenitor subtypes.

The PI3K-AKT signaling pathway is a highly conserved intracellular signal transduction cascade that plays a central role in regulating fundamental cellular processes, including cell growth, proliferation, survival, metabolism, and angiogenesis. The pathway is typically initiated when extracellular ligands bind to receptor tyrosine kinases (RTKs) on the cell surface, triggering receptor dimerization and autophosphorylation. This creates docking sites that recruit PI3K (phosphoinositide 3-kinase) to the plasma membrane. PI3K is a heterodimeric enzyme composed of a regulatory subunit (p85) and a catalytic subunit (p110). Once activated, PI3K catalyzes the phosphorylation of phosphatidylinositol-4,5-bisphosphate (PIP2) to generate phosphatidylinositol-3,4,5-trisphosphate (PIP3). AKT, as a key downstream effector of PI3K, exists in at least three isoforms: AKT1, AKT2, and AKT3. Once activated, AKT exerts broad biological effects primarily by phosphorylating downstream substrates, including mammalian target of rapamycin (mTOR) and GSK3β, thereby promoting protein synthesis, cell growth, and survival. The tumor suppressor gene PTEN acts as a negative regulator of the PI3K-AKT pathway by dephosphorylating PIP3 back to PIP2, thereby terminating downstream signaling [11,12,13].

This study investigated the effects of PI3K and AKT inhibitors on different stages of myocardial differentiation. The results demonstrated that PI3K inhibitors promote early myocardial differentiation and enhance the expression of *Brachyury T*, a hallmark gene of mesodermal differentiation. AKT is stimulated by Wnt signals and AKT inhibitors block the expression of mesodermal differentiation marker genes in the presence or absence of Wnt agonist. Both PI3K and AKT can inhibit late-stage myocardial differentiation and suppress the expression of myocardial contractile genes. Mechanism studies demonstrate that during the mesodermal induction, PI3K inhibitors promote GSK3β inactivation and β-catenin activation, whereas AKT inhibitors exhibit the opposite effect. Transcript sequencing results demonstrated that treatment with a PI3K inhibitor significantly enhanced Wnt3a expression. Therefore, this study demonstrates that the PI3K pathway plays a critical role in maintaining the undifferentiated state of stem cells, and inhibition of the PI3K pathway can promote mesodermal differentiation.

## 2. Materials and Methods

### 2.1. Reagents

DMSO was purchased from Sigma-Aldrich (St. Louis, MO, USA). CHIR, IWP2, DAPT, LY294002, and MK2206 were obtained from TargetMol Chemicals Inc. (Boston, MA, USA). Activin A was purchased from Sino Biological Inc. (Beijing, China). Antibodies against Brachyury T (A5078), β-catenin (A19657), GSK3β (A27666), AKT1 (A11016), p-AKT (AP0140), PI3K p85α (A4992), and Wnt3a (A25322) were purchased from ABclonal Biotechnology (Wuhan, China). The p-GSK3β (9323) antibody used in the present study was obtained from Cell Signaling Technology (Boston, MA, USA). Hoechst 33342, Lipo6000^TM^ Transfection Reagent, ECL Chemiluminescence Detection Kit, BCA Protein Assay Kit, and the Dual-Luciferase Reporter Gene Assay Kit were purchased from Beyotime Biotechnology (Shanghai, China). RNA extraction and real-time quantitative PCR (RT-qPCR) kits were obtained from Vazyme Biotech (Nanjing, China). PCR primers were synthesized by Tsingke Biotechnology (Beijing, China).

### 2.2. P19CL6 Cell Culture and Cardiac Differentiation

Teratoma P19CL6 cells were kindly provided by Dr. Zang (Zhengzhou University) and cultured as reported [14,15]. Undifferentiated P19CL6 cells were maintained in α-MEM medium supplemented with 10% fetal bovine serum (FBS) and 1% penicillin-streptomycin. For cardiac differentiation, cells were cultured in α-MEM containing 10% heat-inactivated FBS, 1% penicillin-streptomycin, and 1% dimethyl sulfoxide (DMSO). All cultures were maintained at 37 °C in a humidified atmosphere of 5% CO_2_. Cardiac differentiation of P19CL6 cells was induced using a two-stage protocol. When cells reached approximately 80% confluence, they were dissociated and seeded at a density of 1.5 × 10^5^ cells per well into low-attachment six-well plates (Corning) for suspension culture. After 4 days of differentiation, the cells formed embryoid bodies (EBs) and were subsequently transferred to adherent culture dishes. During the first 8 days, cells were cultured in differentiation induction medium, after which the medium was replaced with standard culture medium until day 12. For adherent differentiation, P19CL6 cells were seeded directly at a density of 1.5 × 10^5^ cells per well into standard six-well plates and cultured in the presence of DMSO to induce differentiation.

### 2.3. Immunofluorescence

Cells at different stages of differentiation were fixed with 4% paraformaldehyde for 15 min at room temperature. Following fixation, the cells were permeabilized with 0.5% Triton X-100 in PBS (PBST) for 10 min. Non-specific binding sites were blocked by incubation with 2% bovine serum albumin (BSA) for 30 min at 37 °C. Subsequently, the cells were incubated overnight at 4 °C with primary antibodies (1:100 or 1:200) diluted in 2% BSA. After three washes with PBST, the cells were incubated with FITC-conjugated secondary antibody in a humidified chamber for 30 min in the dark. The cells were then washed thoroughly with PBST, and nuclei were counterstained with 1 µg/mL Hoechst 33342 for 5 min. After a final wash, the coverslips were mounted using buffered glycerol (PBS:glycerol = 1:9) and examined under a fluorescence microscope (Leica, Wetzlar, Germany). Positive cell counting was performed using ImageJ software (1.51n) for immunofluorescence sections. Positive signals were identified by uniform threshold segmentation, and regions of interest were manually defined. The software automatically counted positive cells and calculated the ratio of positive cells to total nuclei.

### 2.4. RNA Extraction and Real-Time PCR Analysis

Total RNA was extracted using RNAVzol reagent according to the manufacturer’s instructions (Vazyme Biotech). For real-time quantitative PCR (RT-qPCR), the Taq Pro Universal SYBR qPCR Master Mix (Vazyme Biotech) was employed on an ABI 7700 real-time PCR system. Each 15 µL reaction mixture contained 7.5 µL of 2× Taq Pro Universal SYBR qPCR Master Mix buffer, 0.25 µL each of forward and reverse primers (10 pmol/µL), 1 µL of cDNA template, and nuclease-free deionized water added to bring the final volume to 15 µL. The thermal cycling conditions were as follows: initial denaturation at 95 °C for 10 min, followed by 40 cycles of denaturation at 95 °C for 15 s and annealing/extension at 60 °C for 1 min. The cycle threshold (Ct) values were determined for each sample, and relative gene expression levels between different samples were calculated using the 2^−ΔΔCt^ method. Primer sequences for the tested genes are listed in Table 1. For each condition, 3–4 independent samples were analyzed.

### 2.5. Western Blot Analysis

Cells were washed twice with ice-cold phosphate-buffered saline (PBS), harvested by scraping, and collected into Eppendorf tubes. The cell suspension was centrifuged at 5000 rpm for 5 min, and the resulting cell pellet was resuspended in pre-chilled lysis buffer containing 50 mM Tris-HCl (pH 8.0), 150 mM NaCl, 0.1% SDS, 1% NP-40, and 0.5% sodium deoxycholate. Following incubation on ice for 30 min, the lysate was centrifuged at 12,000 rpm for 15 min, and the supernatant was collected as the whole-cell lysate. Protein concentration was determined using the BCA assay. For each sample, 30 µg of total protein was denatured by boiling and subsequently separated by 10% SDS-PAGE. The separated proteins were then transferred onto a PVDF membrane. After blocking with 5% non-fat dry milk for 1 h, the membrane was incubated with primary antibodies (1:1000 or 1:2000), followed by incubation with horseradish peroxidase (HRP)-conjugated secondary antibodies. Protein bands were visualized using a chemiluminescent detection kit (Beyotime Biotechnology, China).

### 2.6. Transfection

Cell transfection was performed following the manufacturer’s instructions for the Lipo6000^TM^ Transfection Reagent. Briefly, 1.2 μg of pcDNA3 or AKT1 plasmid (purchased from Sino Biological Inc., Beijing, China) was mixed with 2 μL of Lipo6000^TM^ Transfection Reagent (Beyotime Biotechnology) in 50 μL of serum-free α-MEM medium in an Eppendorf tube. The mixture was gently pipetted to ensure thorough mixing and incubated at room temperature for 5 min before being added dropwise to the cells in a 12-well plate. Four hours post-transfection, the medium was replaced with fresh complete medium supplemented with 1% DMSO to induce cardiac differentiation. Cells were cultured for an additional 48 h, after which total RNA was harvested for gene expression analysis.

### 2.7. Luciferase Reporter Experiments

The TOPFlash luciferase reporter plasmid (TOPFlash-Luc), used to monitor Wnt/β-catenin pathway activation, was purchased from Beyotime Biotechnology. A luciferase reporter plasmid driven by the proximal promoter of the *Brachyury T* gene (Brachyury T-Luc) was constructed by Yanjin Biotechnology (Beijing, China). P19CL6 cells were seeded in 12-well plates and transiently transfected with either TOPFlash-Luc or Brachyury T-Luc plasmid (1.2 µg/well) using Lipo6000^TM^ Transfection Reagent (Beyotime Biotechnology). The pRL-TK plasmid (20 ng/well) was co-transfected as an internal control to normalize transfection efficiency. Following transfection, cardiac differentiation was induced by the addition of 1% DMSO. After 24 h, cells were treated with either LY294002 (5 µM) or MK2206 (3 µM) for an additional 24 h. Cells were then harvested, and dual-luciferase activities were measured using the Dual-Luciferase Reporter Gene Assay Kit (Beyotime Biotechnology) according to the manufacturer’s protocol. Luminescence signals were detected using a POLARstar luminometer (Promega, Ortenberg, Germany). Firefly luciferase activity was normalized to renilla luciferase activity. The relative luciferase activity, expressed as the ratio of firefly to renilla luminescence, was used to compare the activation levels of the reporter plasmids across different treatment conditions.

### 2.8. Transcriptome Sequencing and Data Analysis

Total RNA was extracted from cells using the TRIzol reagent (Invitrogen, Waltham, MA, USA) according to the manufacturer’s instructions. The concentration and purity of the RNA samples were assessed using a NanoDrop 2000 spectrophotometer (Thermo Fisher Scientific Inc., Waltham, MA, USA), and RNA integrity was evaluated using an Agilent 2100 Bioanalyzer (Agilent Technologies, Inc., Santa Clara, CA, USA). Only samples with an RNA integrity number (RIN) of 7.0 or higher were considered acceptable for subsequent analyses. Following quality control, qualified RNA samples were submitted to BGI Genomics for transcriptome sequencing (RNA-seq). The raw sequencing reads YE (raw reads) were processed to remove adapter sequences, low-quality reads (those with >20% of bases having a quality score < 20), and reads containing excessive unknown bases (N > 5%). The resulting clean reads were then aligned to the reference genome sequence using HISAT2 software (v2.0.4), with a minimum mapping rate of 80% required. Additionally, Bowtie2 was employed to align the reads to the reference gene sequences, and the expressio n level of each gene was quantified using RSEM software (v1.2.8). Gene expression abundance was presented as fragments per kilobase of transcript per million fragments mapped (FPKM) values. Hierarchical clustering, heatmap visualization, and functional enrichment analyses, including Gene Ontology (GO) and Kyoto Encyclopedia of Genes and Genomes (KEGG) pathway enrichment, were performed using the Dr. Tom online analysis platform provided by BGI Genomics. Raw sequencing data have been deposited in the NCBI BioProject database under accession number PRJNA1531762. The data will be released publicly on 31 December 2026.

### 2.9. Statistics

Each experimental group consisted of a minimum of three biological replicates. Results are expressed as the mean ± standard deviation (SD). Statistical analysis was performed using GraphPad Prism 9 software. The Shapiro–Wilk test was used to evaluate the normality of data, and Levene’s test was used to check homogeneity of variances. One-way ANOVA was adopted for comparison among multiple groups, followed by Tukey’s post hoc test for pairwise multiple comparisons. *p* < 0.05 was considered statistically significant.

## 3. Results

### 3.1. The Establishment of Stepwise Cardiac Differentiation

**Figure 1:**
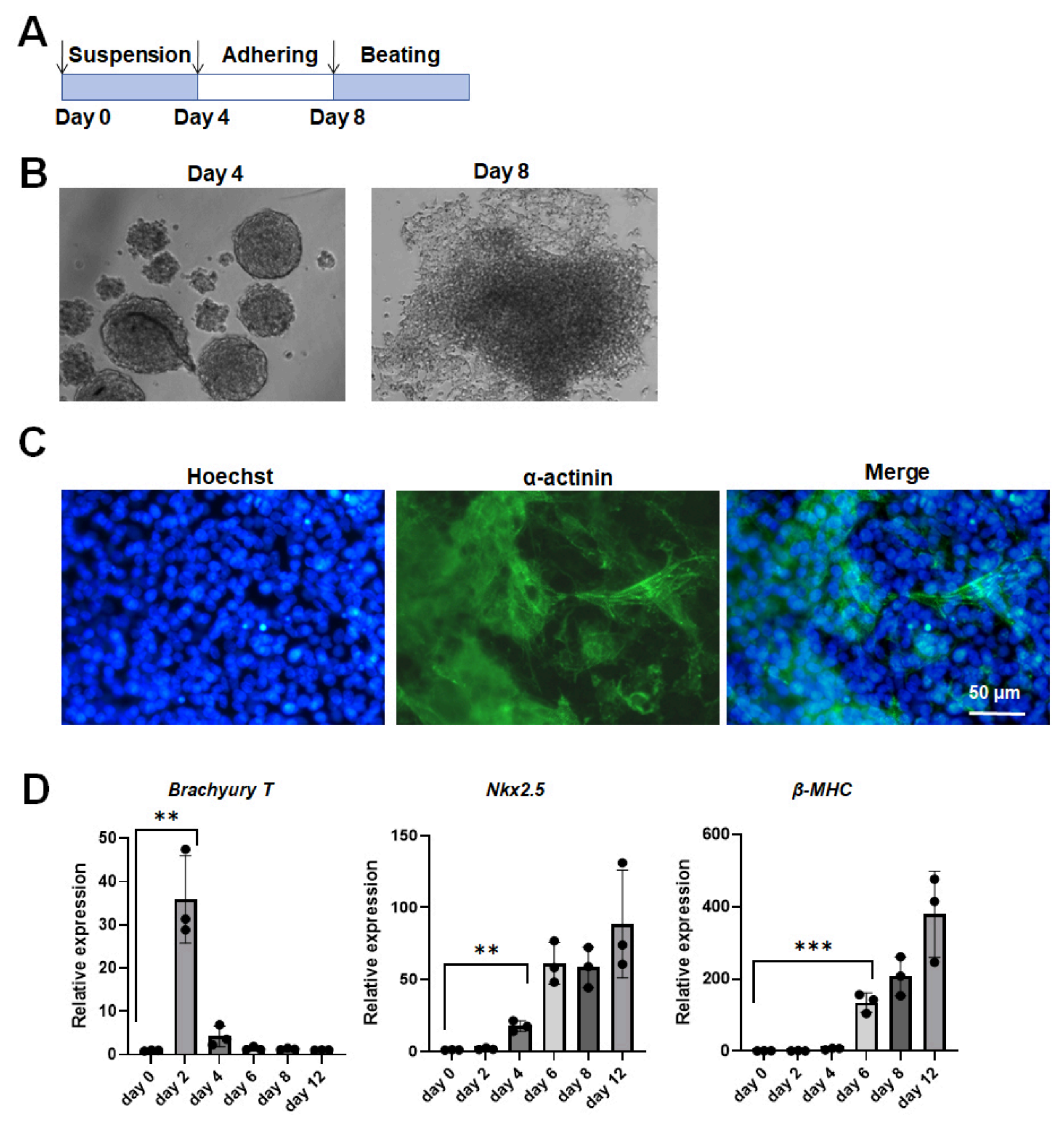
The establishment of two-stage cardiac induction. (**A**) Schematic diagram of differentiation protocol. P19CL6 cells were induced into cardiomyocytes using 1% DMSO with a two-stage induction protocol. (**B**) Morphological changes in cells during induced differentiation, including embryoid body formation and subsequent adherent induction. (**C**) Identification of cardiomyocyte differentiation by α-actinin protein immunofluorescence on day 12. (**D**) The expression of *Brachyury T* (mesodermal marker gene), *Nkx2.5* (cardiac progenitor gene) and *Myl7* (cardiomyocyte functional gene) was detected by real-time PCR. n = 3, ** *p* < 0.01, *** *p* < 0.001.

This study established an in vitro model for the stepwise differentiation of stem cells into cardiomyocytes, wherein dimethyl sulfoxide (DMSO) was used to induce differentiation of P19CL6 mouse teratoma stem cells into cardiomyocytes (Figure 1A). First, P19CL6 cells were cultured in low-adhesion plates to promote cell suspension and aggregation into embryoid bodies (EBs), followed by addition of DMSO to imitate the early development of mammalian embryos. After 4 days, the EBs were transferred to adherent culture dishes for induction. DMSO administration for another 4 days resulted in observable cell spreading and growth. Spontaneous beating myocardial cells were observed from day 10 (Figure 1B). The beating area progressively increased over subsequent days. The beating cells on day 12 were fixed and subjected to immunofluorescence staining to detect the myocardial mechanical contraction protein α-actinin (Figure 1C). The results demonstrate that differentiated cells express α-actinin and form sarcomere structure.

Real-time PCR was performed to detect the expression of stage-specific genes during cardiac differentiation. The results showed that the mesodermal marker gene *Brachyury T* was highly expressed only on day 2 of differentiation, but its expression markedly decreased by day 4, exhibiting a transient expression pattern. In contrast, the cardiac transcription factor *Nkx2.5* began to be expressed on day 4 and persisted. The functional gene for myocardial mechanical contraction, myosin light chain 7 (*Myl7*), started to be expressed during the adhesion induction stage and was significantly upregulated by day 12, correlating with the initiation of spontaneous cardiomyocyte beating. These results collectively demonstrate that myocardial differentiation proceeds through distinct stage-specific phases (Figure 1D). The above experiments demonstrate that myocardial-induced differentiation has been successfully established.

### 3.2. The Expression Regulation of Mesodermal Differentiation Marker Gene

**Figure 2:**
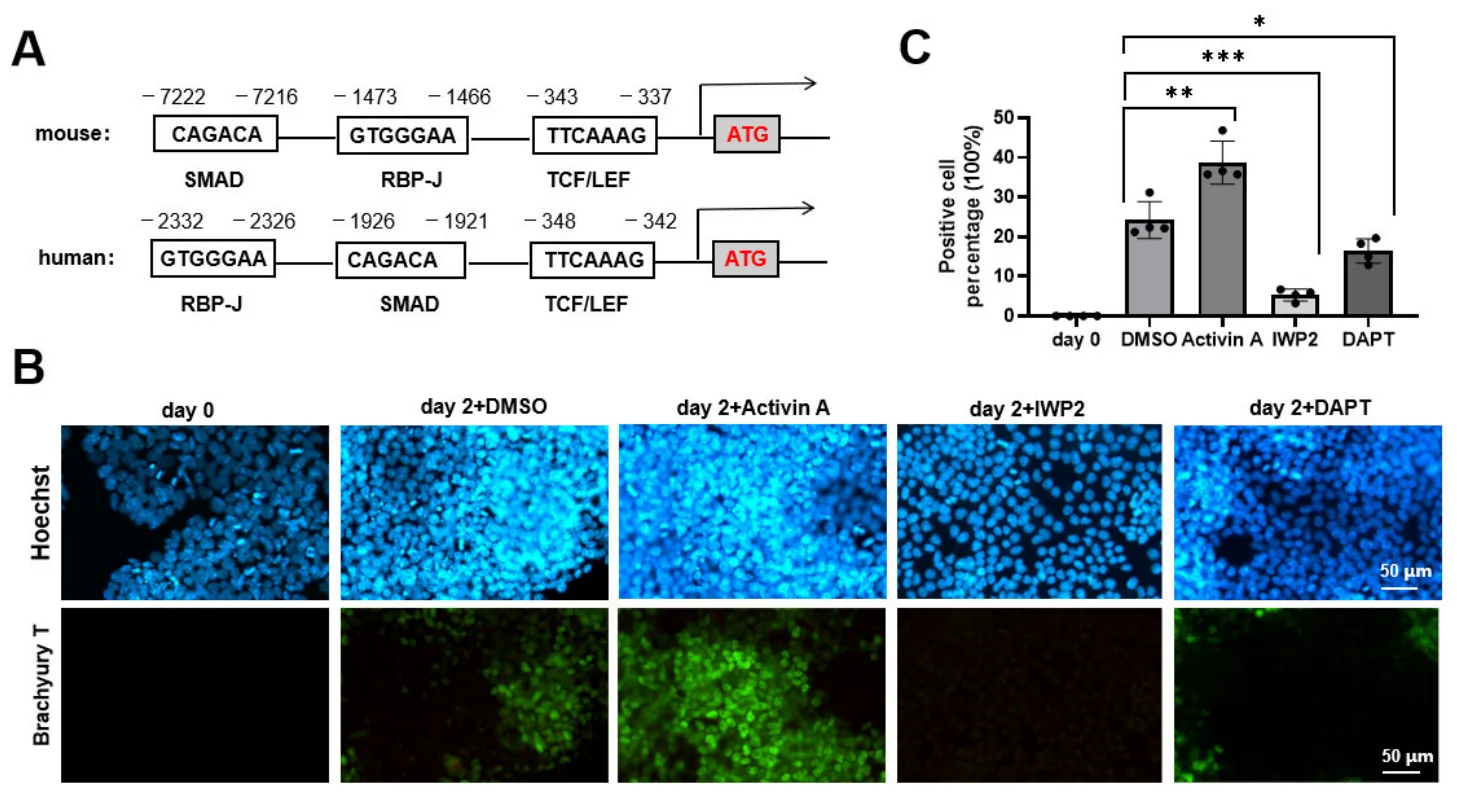
The expression regulation of the *Brachyury T* gene. (**A**) The comparison and analysis of the proximal *Brachyury T* promoters between human and mouse. (**B**) P19CL6 cells were treated with 50 ng/mL Activin A, 5 µM IWP2, or 5 µM DAPT for 2 days under adherent induction conditions, and the expression of Brachyury T protein was detected by immunofluorescence. (**C**) The positive cell rate was derived from statistical analysis of four randomly selected fields of view. n = 4, * *p* < 0.05, ** *p* < 0.01, *** *p* < 0.001.

Brachyury T is a well-established marker gene for mesodermal differentiation. Here, we analyzed the promoter region of the *Brachyury T* gene. The proximal promoter of *Brachyury T* is conserved between humans and mice and contains a “TTCAAAG” sequence. The complementary strand of this sequence conforms to the binding motif of TCF/LEF transcription factors, thereby enabling responsiveness to Wnt signaling. In addition, the proximal promoter and intronic regions of the *Brachyury T* gene harbor binding sites for SMAD transcription factors, which mediate responsiveness to Activin/Nodal signaling. Furthermore, the proximal promoters of both human and mouse *Brachyury T* genes contain a “GTGGGAA” sequence, which serves as a binding site for the transcription factor RBP-J, thereby mediating responsiveness to Notch signaling (Figure 2A).

Next, we investigated the effects of modulating different signaling pathways on Brachyury T expression. Immunofluorescence experiments demonstrated that undifferentiated P19CL6 cells do not express Brachyury T. Cell differentiation was initiated by DMSO induction, and the Brachyury T protein expressed in the cell nucleus could be detected on the second day of differentiation. Treatment with Activin A significantly increased the number of Brachyury T-positive cells, indicating that the Activin/Nodal signaling pathway promotes Brachyury T expression. Treatment of cells with the Wnt pathway inhibitor IWP2 completely suppressed Brachyury T expression, indicating that Wnt activation is essential for Brachyury T expression. Treatment of cells with the Notch pathway inhibitor DAPT significantly suppressed Brachyury T expression, further demonstrating that the Notch pathway positively regulates Brachyury T expression (Figure 2B,C). Overall, this study confirms that the expression of the mesodermal marker gene Brachyury T is regulated by multiple signaling pathways.

### 3.3. The Different Effects of PI3K and AKT Inhibitors on Mesodermal Differentiation

**Figure 3:**
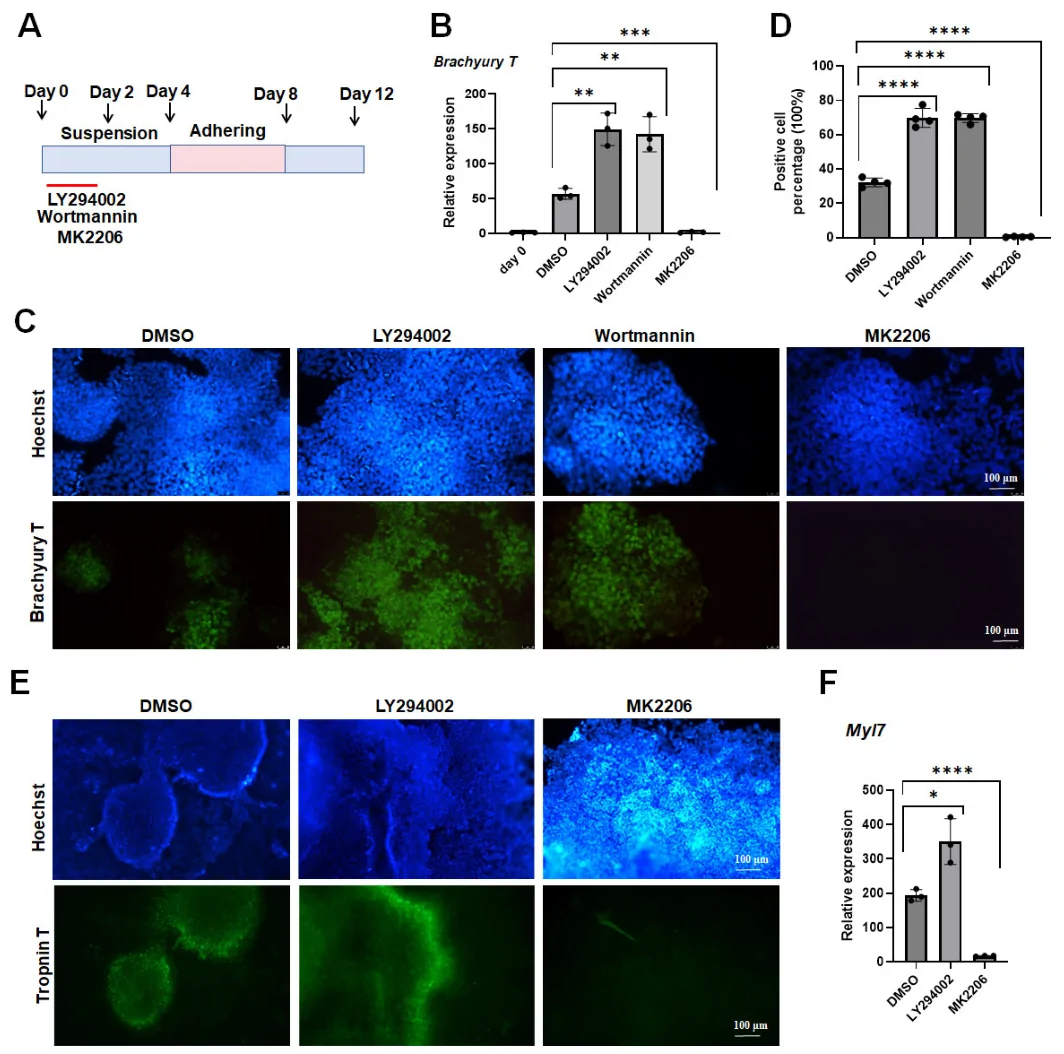
PI3K and AKT inhibitors differently affect mesodermal differentiation. (**A**) Schematic diagram of PI3K and AKT inhibition on mesodermal differentiation. (**B**) P19CL6 cells were induced by 1% DMSO in low-adhesion plates to form EBs. At the same time, the EBs were cultured with LY294002 (5 µM), Wortmannin (5 µM), or MK2206 (3 µM) for 2 days. Real-time PCR was performed to detect the expression of the *Brachyury T* gene. n = 3, ** *p* < 0.01, *** *p* < 0.001. (**C**,**D**) P19CL6 cells were induced by 1% DMSO under adherent induction conditions. At the same time, the cells were cultured with LY294002 (5 µM), Wortmannin (5 µM), or MK2206 (3 µM) for 2 days. The expression of the Brachyury T protein was detected by immunofluorescence on day 2. The Brachyury T-positive cell rate percentage was derived from statistical analysis of four randomly selected fields of view. n = 4, **** *p* < 0.0001. (**E**) P19CL6 cells were induced with a two-stage induction protocol and were treated with PI3K or AKT inhibitors on mesodermal differentiation. Troponin T was detected by immunofluorescence analysis on day 12. (**F**) After the treatment of PI3K or AKT inhibitors, cells were subjected to further induction and culture. The expression of *Myl7* was detected by real-time PCR on day 12. n = 3, * *p* < 0.05, **** *p* < 0.0001.

We next examined the effects of PI3K and AKT pathways on early myocardial differentiation (Figure 3A). Inhibition of the PI3K pathway using LY294002 significantly enhanced the mRNA expression of the mesodermal differentiation marker *Brachyury T*. In contrast, inhibition of the AKT pathway with MK2206 nearly completely abolished the expression of *Brachyury T* mRNA. Another PI3K pathway inhibitor, Wortmannin, produced effects similar to those of LY294002, promoting *Brachyury T* expression (Figure 3B). To further validate these findings, P19CL6 cells were subjected to adherent differentiation culture and subsequently analyzed by immunofluorescence for Brachyury T protein expression (Figure 3C,D). Both LY294002 and Wortmannin markedly increased the number of Brachyury T-positive cells on day 2 of differentiation, whereas MK2206 completely blocked Brachyury T expression. These results demonstrate that inhibition of the PI3K pathway positively regulates mesodermal differentiation, while inhibition of AKT exerts a negative regulatory effect at this early stage.

Subsequently, we examined the effects of early-stage treatment with PI3K and AKT inhibitors on terminal cardiomyocyte differentiation. Treatment with the PI3K inhibitor LY294002 during the first two days of differentiation significantly enhanced the efficiency of terminal cardiomyocyte differentiation. As demonstrated by immunofluorescence analysis on day 12 of differentiation, LY294002 increased the expression of the cardiac contractile protein Troponin T. In contrast, the AKT inhibitor MK2206 nearly completely abolished the emergence of functional cardiomyocytes expressing Troponin T (Figure 3E). Similarly, real-time PCR analysis revealed that LY294002 upregulated the expression of *Myl7*, whereas MK2206 completely blocked its expression (Figure 3F). Collectively, these results indicate that PI3K and AKT pathway inhibitors exert differential regulatory effects on early cardiomyocyte differentiation, thereby modulating the efficiency of terminal cardiomyocyte differentiation.

### 3.4. Wnt-Mediated AKT Activation Favors Mesodermal Differentiation

**Figure 4:**
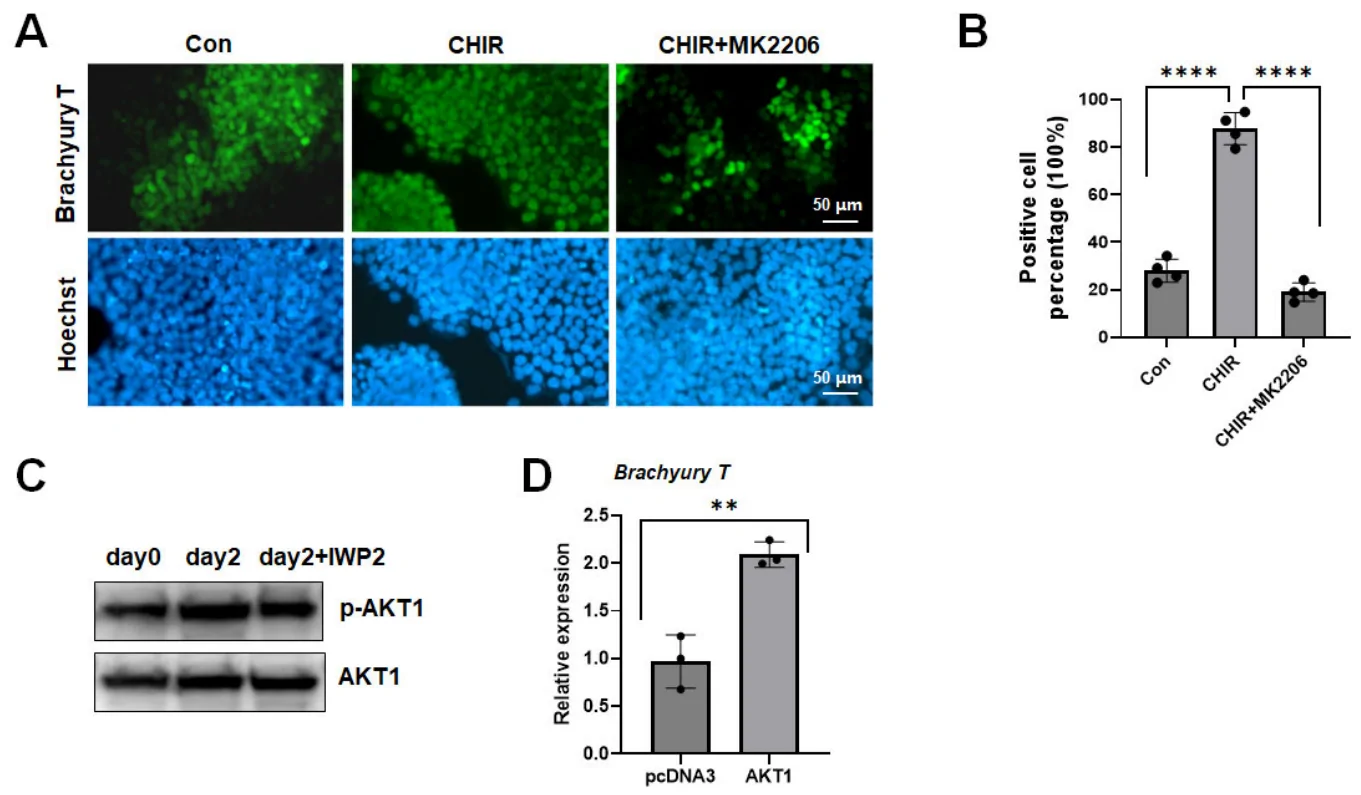
Wnt-mediated AKT activation is required for mesodermal differentiation. (**A**,**B**) P19CL6 cells were induced by 1% DMSO under adherent induction conditions. At the same time, the cells were cultured with CHIR (2.5 µM) or CHIR+MK2206 (3 µM) for 2 days. The expression of the Brachyury T protein was detected by immunofluorescence on day 2. The Brachyury T-positive cell rate percentage was derived from statistical analysis of four randomly selected fields of view. n = 4, **** *p* < 0.0001. (**C**) The phosphorylated AKT levels in cells treated with either normal induction or IWP2 were measured. (**D**) P19CL6 cells were transfected with pcDNA3 or AKT1 plasmids and then subjected to cardiac induction for 2 days. Real-time PCR was performed to detect the expression of the *Brachyury T* gene. n = 3, ** *p* < 0.01.

When we used the small-molecule inhibitor CHIR to activate Wnt signaling, we observed enhanced mesodermal differentiation as evidenced by the staining of Brachyury T. However, the effect of CHIR was greatly abolished by the addition of MK2206 (Figure 4A,B). Our further studies demonstrated that mesodermal differentiation promoted AKT phosphorylation. However, treatment with IWP2 to antagonize the Wnt pathway effectively inhibited AKT phosphorylation and activation (Figure 4C, see Supplementary Materials). We further use transient transfection to overexpress the AKT1 gene in P19CL6 cells. As a result, AKT1 overexpression enhanced the expression of Brachyury T (Figure 4D). Therefore, our finding implicated that the Wnt pathway can promote AKT activation, and the AKT pathway favors mesodermal differentiation.

### 3.5. The Influence of PI3K and AKT Inhibitors on the Late-Stage Cardiac Differentiation

**Figure 5:**
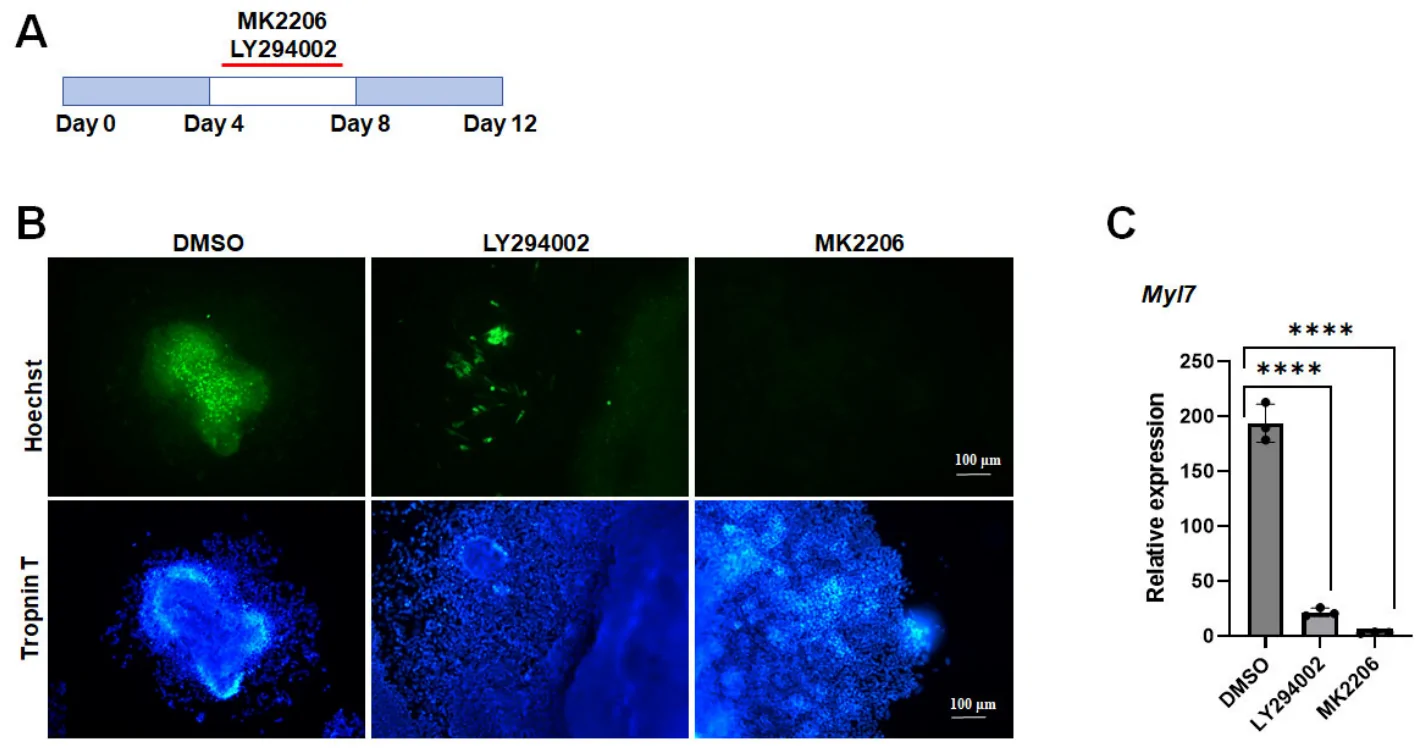
PI3K and AKT inhibitors affect the late-stage cardiac differentiation. (**A**) Schematic diagram of PI3K (LY294002, 10 µM) and AKT (MK2206, 6 µM) inhibition on the late-stage cardiac differentiation. (**B**) Troponin T was detected by immunofluorescence analysis on day 12. (**C**) After the treatment of PI3K or AKT inhibitors, cells were subjected to further induction and culture. The expression of *Myl7* was detected by real-time PCR on day 12. n = 3, **** *p* < 0.0001.

We next investigated the effects of PI3K and AKT inhibitors on late-stage myocardial differentiation. As illustrated in Figure 5A, P19CL6 cells were first subjected to suspension culture, and then EBs were plated for adherent differentiation, during which PI3K or AKT inhibitors were added. Immunofluorescence analysis revealed that both PI3K and AKT inhibitors effectively suppressed the expression of the cardiac contractile protein Troponin T (Figure 5B). Similarly, real-time PCR assays demonstrated that LY294002 and MK2206 inhibited the expression of the myocardial differentiation marker gene *Myl7* (Figure 5C). Collectively, these results indicate that both PI3K and AKT inhibitors exert comparable inhibitory effects on late-stage myocardial differentiation.

### 3.6. The Modulation of PI3K and AKT Inhibitors on GSK3β and β-Catenin Activation

It is well known that the Wnt/β-catenin signaling pathway plays a critical role in mesodermal differentiation, and GSK3β inhibits β-catenin activation through phosphorylation, thereby promoting its degradation. While AKT kinase can phosphorylate and inhibit GSK3β activity. So, we next investigated whether PI3K and AKT inhibitors differentially regulate mesodermal differentiation by modulating GSK3β and β-catenin.

**Figure 6:**
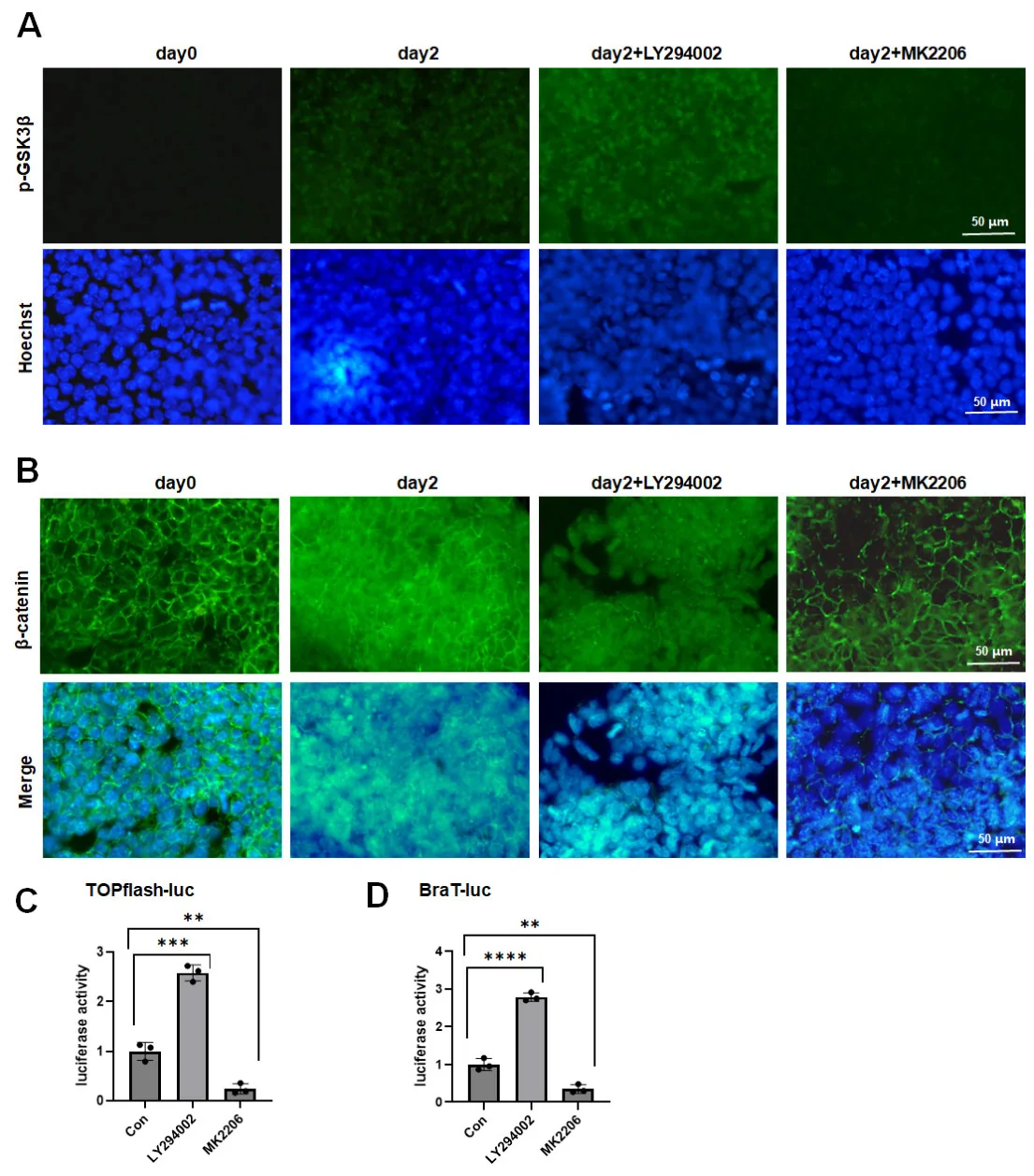
PI3K and AKT inhibitors modulate GSK3β and β-catenin activation. (**A**) P19CL6 cells were induced by 1% DMSO under adherent induction conditions to initiate mesodermal differentiation. After the treatment of PI3K or AKT inhibitors for 2 days, cells were immunostained with antibodies against phosphorylated GSK3β (green). The nuclei were stained with DAPI (blue). (**B**) β-catenin was detected by immunofluorescence analysis on day 2. The images are representative of results obtained from two independent replicate experiments. (**C**,**D**) TOPflash-luc or *Brachyury T-*luc plasmids were overexpressed in P19CL6 cells with pRL-TK as the internal reference plasmid. Then cells were subjected to cardiac induction and inhibitor treatment for 2 days. Cells were harvested, and luciferase activity was detected. n = 3, ** *p* < 0.01, *** *p* < 0.001, **** *p* < 0.0001.

As shown in Figure 6A, the level of phosphorylated GSK3β (p-GSK3β) in the cytoplasm was significantly increased upon differentiation. Treatment with the PI3K inhibitor LY294002 markedly enhanced GSK3β phosphorylation (Ser9). In contrast, the AKT inhibitor MK2206 substantially suppressed GSK3β phosphorylation. Furthermore, in undifferentiated cells, β-catenin was predominantly localized beneath the plasma membrane, likely participating in cell junction formation through association with cadherins. Upon differentiation, β-catenin translocated from the submembrane region to the cytoplasm, accumulated in the cytosol, and subsequently entered the nucleus. The LY294002 treatment reduced the submembrane localization of β-catenin and significantly enhanced their nuclear translocation. In contrast, MK2206 markedly decreased the cytoplasmic content of β-catenin and maintained its submembrane localization (Figure 6B).

Using the TOPFlash luciferase reporter plasmid to assess Wnt/β-catenin pathway activation, we found that treatment with LY294002 significantly enhanced reporter activity, whereas treatment with MK2206 markedly suppressed Wnt pathway activation (Figure 6C). Similarly, employing a luciferase reporter plasmid to monitor the endogenous *Brachyury T* gene promoter activity, we observed that the PI3K inhibitor increased promoter activity, while the AKT inhibitor substantially reduced it (Figure 6D). Collectively, these results demonstrate that PI3K and AKT inhibitors differentially regulate the GSK3β and Wnt/β-catenin pathway during early myocardial differentiation, thereby exerting opposing effects on mesodermal commitment.

### 3.7. The Effect of PI3K and AKT Inhibitors on the Localization of AKT1, GSK3β, and the PI3K p85 Subunit

**Figure 7:**
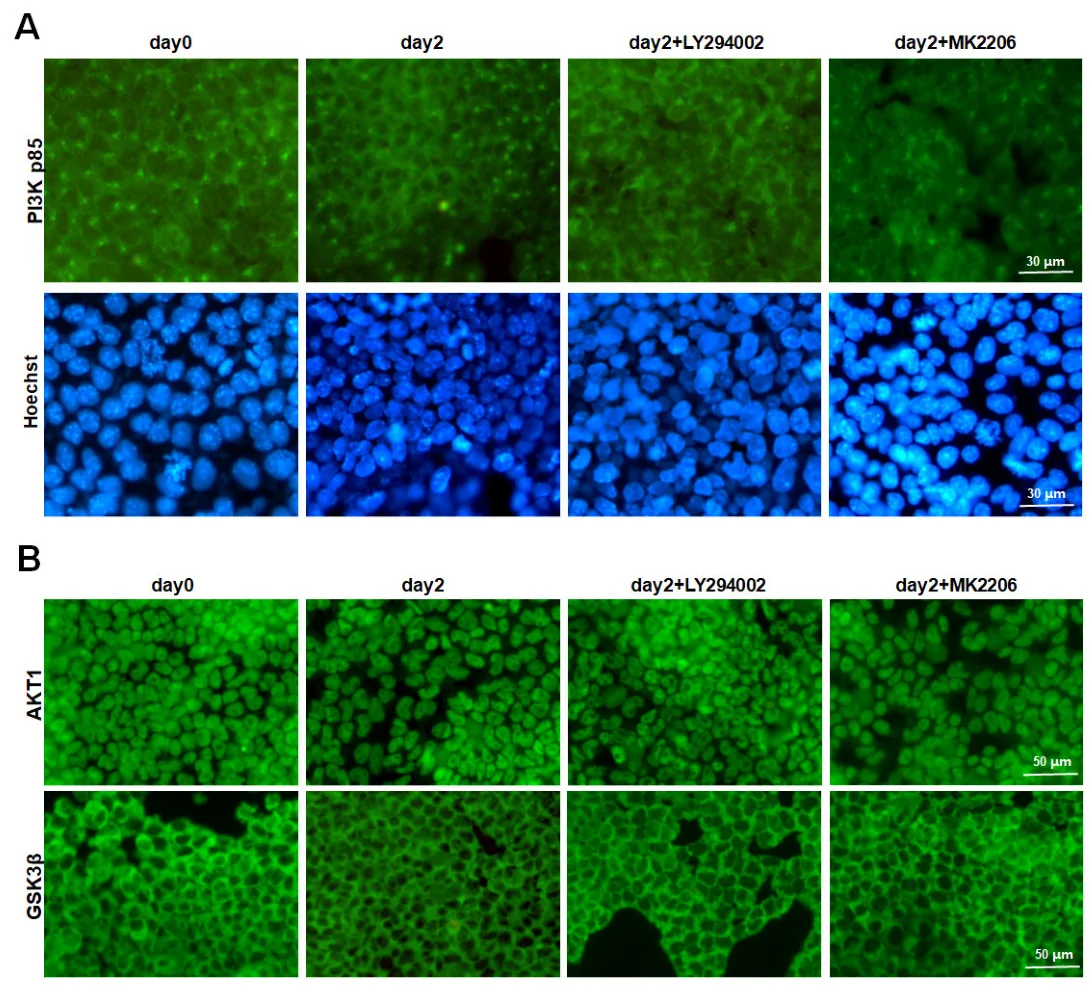
PI3K and AKT inhibitors modulate the localization of AKT1, GSK3β, and PI3K p85 subunit. (**A**) P19CL6 cells were induced by 1% DMSO under adherent induction conditions to initiate mesodermal differentiation. After the treatment of PI3K or AKT inhibitors for 2 days, cells were immunostained with antibodies against PI3K p85α subunit (green). The nuclei were stained with DAPI (blue). (**B**) AKT1 and GSK3β were detected by immunofluorescence analysis on day 2. The images are representative of results obtained from two independent replicate experiments.

PI3K is a heterodimeric enzyme composed of a regulatory subunit (p85) and a catalytic subunit (p110). Next, we examined the subcellular localization changes in the PI3K p85α subunit by immunofluorescence assay (Figure 7A). The results showed that in undifferentiated cells, the PI3K p85α subunit was localized in the cytoplasm and exhibited distinct punctate aggregation signals. Upon differentiation, the p85 subunit became diffusely distributed throughout the cytoplasm. Treatment with LY294002 markedly enhanced this diffuse distribution pattern. In contrast, treatment with MK2206 preserved the punctate aggregation signals of p85α. In addition, AKT1 was consistently localized in the nucleus both before and after differentiation, while GSK3 remained persistently localized in the cytoplasm, with neither exhibiting significant changes in subcellular localization throughout the differentiation process (Figure 7B). These observations suggest that the PI3K pathway undergoes significant changes in both activity and subcellular localization before and after differentiation, potentially participating in stem cell self-renewal and directed differentiation, respectively.

### 3.8. PI3K Inhibition Affects Wnt, Notch, and Activin/Nodal Signals

**Figure 8:**
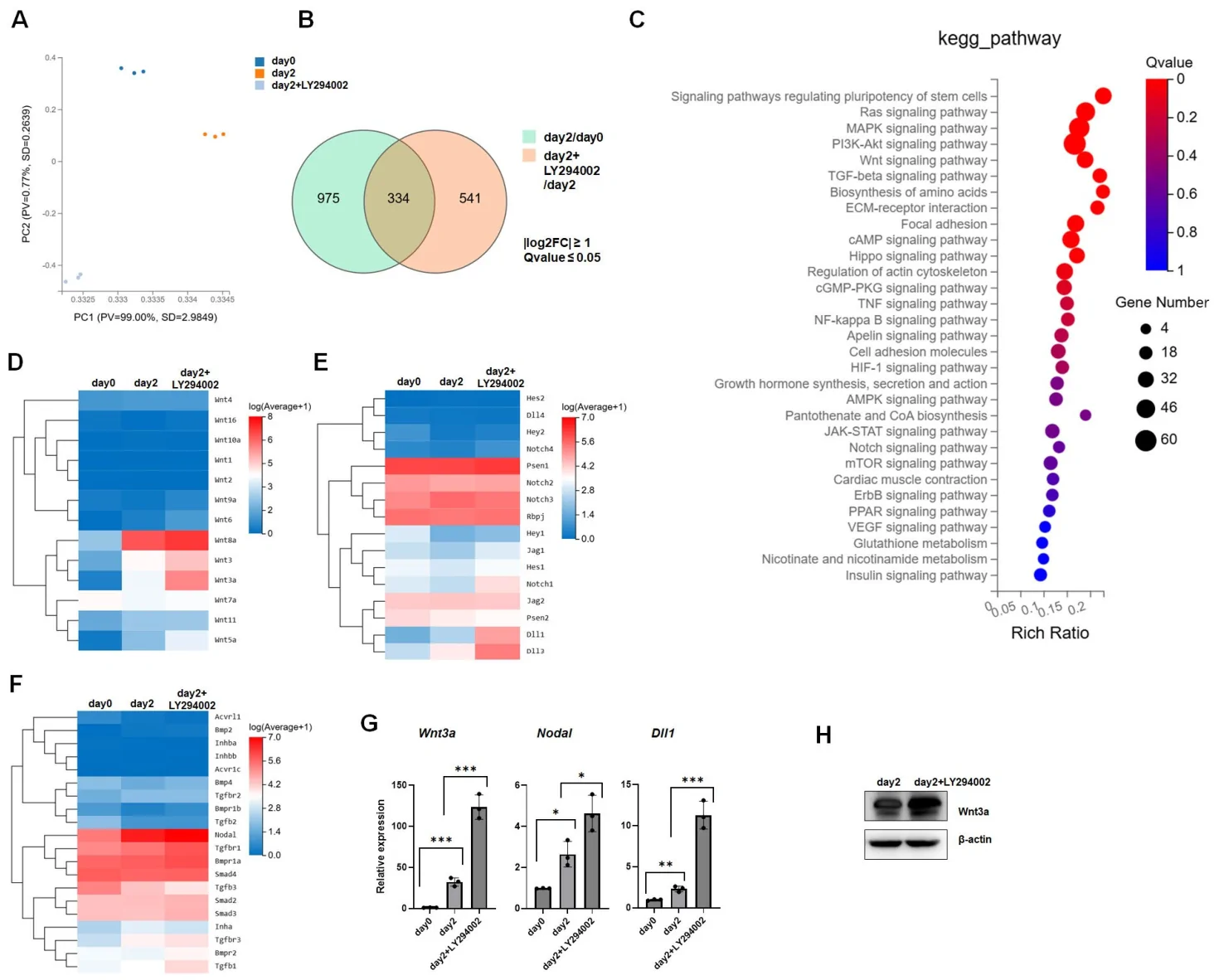
PI3K inhibition modulates Wnt, Notch, and Activin/Nodal signals. (**A**) P19CL6 cells were induced by 1% DMSO under adherent induction conditions to initiate mesodermal differentiation. After the treatment of LY294002 (5 µM) for 2 days, cells were used for transcriptome sequencing. PCA plot showing the transcriptional similarity and biological replicates of samples. (**B**) Venn analysis of differentially expressed genes among three groups (|log2FC| ≥ 1, Q-value ≤ 0.05). (**C**) KEGG enrichment analysis of 334 overlapping genes. (**D**–**F**) Heatmap and clustering analysis of genes associated with Wnt, Notch, and Activin/Nodal signaling pathways. Normalized gene expression levels are presented as the average TPM (Transcripts Per Kilobase Million) values of genes across three biological replicates. (**G**) Real-time PCR analysis was performed to evaluate the effect of LY294002 treatment on *Wnt3a*, *Nodal*, and *Dll1* expression. n = 3, * *p* < 0.05, ** *p* < 0.01, *** *p* < 0.001. (**H**) Western blot was used to detect the protein expression of Wnt3a.

The expression of mesodermal marker gene Brachyury T is regulated by Wnt, Notch, and Activin/Nodal signaling pathways. We next performed transcriptome sequencing (RNA-seq) to investigate the effects of PI3K inhibition on these signaling pathways during cardiac differentiation. Principal component analysis (PCA) based on transcriptomic profiles was performed to assess the intergroup variation and intragroup reproducibility. Samples from three groups (day0, day2, and day2+LY294002) were clearly separated along the PC1 axis, which explained 99.00% of the total variance. Biological replicates within each group clustered tightly together, demonstrating good repeatability of the RNA-seq data and distinct transcriptional differences across the three experimental groups (Figure 8A). Venn diagram analysis of transcriptomic differential gene expression revealed that 1309 genes were significantly differentially expressed in cells after 2 days of induced differentiation compared with undifferentiated cells. In the comparison between LY294002-treated cells and cells undergoing 2 days of differentiation, 875 genes exhibited significant expression changes. The intersection of these two gene sets contained 334 genes (Figure 8B). KEGG pathway enrichment analysis of these 334 overlapping genes demonstrated that the differentially expressed genes were involved in multiple signaling pathways, including the Wnt, TGF-β, and Notch pathways (Figure 8C). The results revealed that upon differentiation of P19CL6 cells, the expression levels of *Wnt3*, *Wnt3a*, and *Wnt8* were significantly upregulated. Notably, treatment with the PI3K inhibitor LY294002 further enhanced the expression of these Wnt ligands (Figure 8D). Furthermore, PI3K inhibition markedly elevated the expression of several genes involved in the Notch signaling pathway, including *Notch1*, *Dll1*, and *Dll3* (Figure 8E). Following the initiation of differentiation, the expression of *Nodal* and *Tgfbr1* was increased, and LY294002 treatment further elevated their expression levels. However, PI3K inhibition had little effect on the expression of *activin A* (*Inhba*) (Figure 8F). The enhanced expression of *Wnt3a, Nodal*, *and Dll1* upon PI3K pathway inhibition was further validated by real-time PCR and immunoblotting analyses (Figure 8G,H, see Supplementary Materials). Collectively, these findings indicate that suppression of the PI3K pathway may promote mesodermal differentiation by coordinately regulating the Wnt, Notch, and Nodal signaling pathways.

### 3.9. PI3K Inhibition Affects the Expression of Genes Associated with Stem Cell Renewal, Mesodermal Differentiation, and PI3K Pathway Modulation

**Figure 9:**
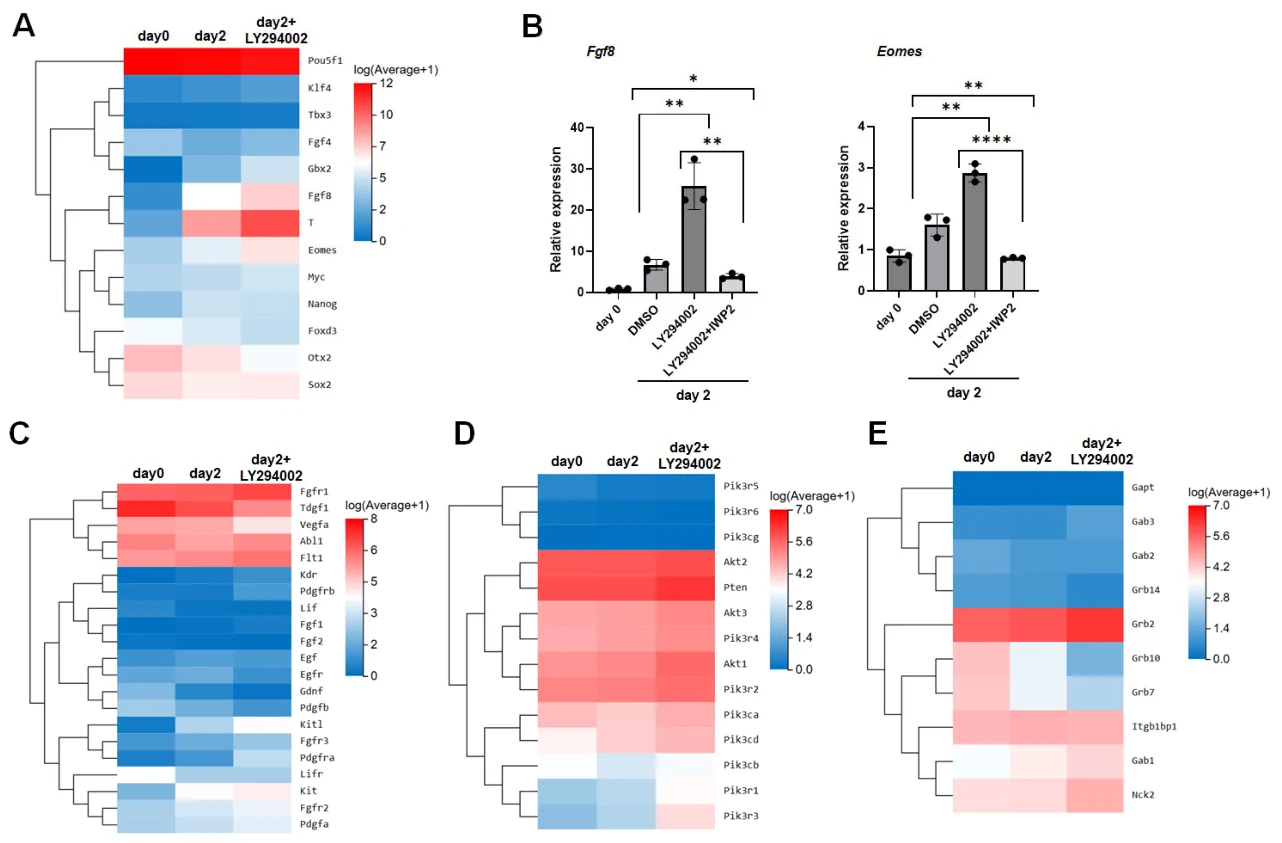
Clustering analysis for genes associated with stem cell renewal, mesodermal differentiation, and PI3K pathway modulation. (**A**) Heatmap and clustering analysis of genes associated with stem cell renewal and mesodermal differentiation. (**B**) After treatment of LY294002 (5 µM) alone or together with IWP2 (5 µM) for 2 days under induction conditions, real-time PCR was performed to detect the expression of the *Fgf8* and *Eomes* genes. * *p* < 0.05, ** *p* < 0.01, **** *p* < 0.0001. (**C**) Clustering analysis of growth factor and receptor genes. (**D**) Expression analysis of PI3K-AKT pathway genes. (**E**) Expression analysis of growth factor receptor-bound adapter genes.

We next examined whether PI3K inhibitor treatment affects the expression of pluripotency-related genes. The results showed that upon initiation of cardiac differentiation, mesodermal marker genes including *Brachyury T* (*T*), *Fgf8*, and *Eomes* were significantly upregulated, and PI3K inhibitor treatment further enhanced the expression of all three genes (Figure 9A). Inhibition of the PI3K pathway using LY294002 significantly enhanced the mRNA expression of mesodermal markers *Fgf8* and *Eomes*. Inhibition of the Wnt pathway with IWP2 nearly completely abolished the upregulation of these two genes (Figure 9B). This finding implicates that Wnt activation is required for PI3K inhibition to favor the expression of mesodermal marker genes.

In contrast, both differentiation induction and PI3K inhibitor treatment had relatively minor effects on the expression of pluripotency-associated genes, with *Oct4* (*Pou5f1*), *Sox2*, *Nanog*, and *Klf4* showing only modest changes (Figure 9A). These findings suggest that PI3K pathway inhibition specifically promotes mesodermal lineage commitment without substantially altering the core pluripotency network.

We next analyzed the regulatory effects of PI3K inhibitor treatment on the PI3K-AKT signaling pathway. First, we observed that upon the initiation of cardiac differentiation, the expression levels of several growth factors and their receptors were significantly altered. Specifically, the expression of *Kit* and its ligand *Kitl* (also known as *Scf*) were markedly upregulated, whereas the expression of *Tdgf1* (teratocarcinoma-derived growth factor 1) and *Lif* (leukemia inhibitory factor) were significantly decreased following differentiation and further suppressed by PI3K inhibitor treatment (Figure 9C).

Second, we found that several components of the PI3K-AKT pathway were modulated by both cardiac differentiation and PI3K inhibitor treatment, most notably *Pik3r1* (*p85α*) and *Pik3r3*, which encode the regulatory subunits of PI3K (Figure 9D). Third, we observed significant changes in the expression of intracellular adaptor proteins of the PI3K-AKT pathway before and after differentiation. In particular, *Grb7* and *Grb10* were downregulated upon differentiation, and PI3K inhibitor treatment further suppressed their expression (Figure 9E). Collectively, these findings suggest that upon initiating the cardiac differentiation program, stem cells may modulate both upstream and downstream components as well as intracellular signaling mediators of the PI3K-AKT pathway, thereby influencing endogenous PI3K and AKT kinase activity to promote mesodermal differentiation. PI3K pathway inhibition also alters the expression of these genes, facilitating the exit from pluripotency maintenance and entry into the differentiation program.

## 4. Discussion

This study investigated the effects and underlying mechanisms of PI3K and AKT inhibitors on cardiomyocyte differentiation. The results revealed that PI3K and AKT inhibitors exert distinct roles during early cardiomyocyte differentiation: PI3K inhibition promoted mesoderm differentiation, whereas AKT inhibition completely blocked it. However, both inhibitors disrupted late-stage cardiomyocyte differentiation. Mechanistic analysis indicated that PI3K inhibition facilitates mesoderm differentiation by modulating signaling pathways such as Wnt, Activin/Nodal, and Notch. Furthermore, the pro-mesodermal effect of PI3K inhibition may be attributed to the suppression of genes associated with stem cell self-renewal, such as *Grb7*.

Since PI3K and AKT kinases function in a sequential upstream-downstream relationship within the same signaling cascade, PI3K inhibitors and AKT inhibitors are generally expected to exert consistent pharmacological effects to regulate diverse cell events under normal circumstances. The PI3K-AKT signaling pathway confers cardioprotective effects through a multi-target mechanism, involving the suppression of inflammation and apoptosis, attenuation of oxidative stress, inhibition of myocardial fibrosis, promotion of angiogenesis, and modulation of autophagy [11,12,13]. Human ES-derived cardiovascular progenitor cells (hCVPCs) promote cardiac repair following myocardial infarction by activating the PI3K-AKT signaling pathway in host cardiomyocytes via a paracrine mechanism, thereby inducing cardiomyocyte dedifferentiation and cell cycle re-entry [16].

Previous studies have demonstrated that the PI3K-AKT pathway serves as a central hub in stem cell differentiation into cardiomyocytes. miR-301a promotes cardiomyocyte differentiation of mouse ES cells by directly targeting and suppressing PTEN, a negative regulator of the PI3K-AKT signaling pathway. This suppression leads to the activation of the PI3K-AKT-mTOR-STAT3 signaling cascade, which in turn upregulates the expression of cardiac transcription factors, including GATA-4, TBX5, and MEF2C, and facilitates the formation of beating cardiomyocyte clones [17]. In iPSCs, the upregulation of NRG1/ErbB4 expression promotes differentiation toward cardiomyocytes by activating the PI3K-AKT signaling pathway. This subsequently leads to the upregulation of cardiac markers, including ANP, NKX2.5, and GATA-4 at the transcriptional level, as well as increased protein expression of α-MHC and cTnT. Notably, this pro-differentiation effect can be significantly reversed by treatment with the PI3K-specific inhibitor LY294002 [18].

It is noteworthy that the PI3K-AKT signaling pathway exhibits a phase-dependent downregulation during cardiomyocyte maturation. Transcriptomic and epigenetic analyses have revealed that multiple components of this pathway are markedly downregulated in the postnatal human heart. In line with this observation, dual inhibition of the MAPK and PI3K/AKT pathways in differentiated human iPSC-derived cardiomyocytes (hiPSC-CMs) for only 5 days significantly enhances several key maturation parameters, including hypertrophy, multinucleation, metabolic shift, T-tubule density, calcium handling, and electrophysiological properties, achieving a maturation state comparable to that of day 60 hiPSC-CMs. These findings collectively indicate that suppression of the PI3K-AKT pathway is a prerequisite for driving terminal cardiomyocyte maturation [19].

However, the role of the PI3K-AKT signaling pathway in mesoderm differentiation remains a subject of debate. Activated PI3K-AKT signal promotes mesoderm fate commitment in mouse ES cells, as evidenced by the upregulation of *Brachyury* expression, while simultaneously suppressing differentiation toward ectoderm and endoderm lineages [20]. In the P19CL6 cell differentiation model, the PI3K-AKT signaling pathway drives early cardiomyogenesis by suppressing GSK3β activity, thereby maintaining the activation of the Wnt/β-catenin signaling cascade. Inhibition of the PI3K-AKT pathway leads to the suppression of Wnt/β-catenin signaling and consequently impairs cardiomyocyte differentiation. Conversely, forced expression of a non-phosphorylated, constitutively active form of β-catenin is sufficient to rescue this differentiation defect. These findings collectively indicate that the PI3K pathway regulates early cardiomyocyte differentiation through a GSK3β/Wnt/β-catenin-dependent mechanism [21].

Paradoxically, several studies have reported that inhibition of the PI3K-AKT pathway can promote myocardial differentiation. Treatment with PI3K inhibitors, such as LY294002, significantly enhances the efficiency of Activin A-induced differentiation toward mesendoderm and definitive endoderm in human ES cells. This process is mechanistically dependent on the prolonged duration of Smad2/3 signaling activity following PI3K inhibition [22]. A separate study demonstrated that, in human iPS cells, the PI3K-AKT signaling pathway maintains the pro-self-renewal function of Activin A/Smad2,3 by suppressing pro-differentiation signals, the Raf/MEk/ERK and canonical Wnt pathways. When PI3K-AKT signaling is reduced, ERK activates the canonical Wnt pathway, which triggers a functional switch in Smad2/3 from a promoter of self-renewal to a promoter of differentiation. In this differentiated state, Smad2/3 collaborates with β-catenin to activate mesendoderm-specific genes [23]. Another study has demonstrated that the small molecule LY294002 significantly promotes cardiomyocyte differentiation by inhibiting the IGF signaling pathway, while simultaneously suppressing the generation of epicardial cells and non-cardiac cell types, including endothelial and mesenchymal cells [24].

Herein, our study contributes to elucidating the role of the PI3K-AKT pathway in myocardial differentiation and partially resolves previously contradictory findings. We found that both PI3K and AKT inhibitors suppress late-stage cardiomyocyte differentiation, suggesting that the PI3K-AKT signaling pathway is essential for this later phase. However, these inhibitors exert strikingly different effects on early-stage cardiomyocyte differentiation. Specifically, the PI3K inhibitors LY294002 and Wortmannin both significantly promote mesoderm differentiation, whereas AKT inhibitors almost completely block it. Mechanistic studies revealed that PI3K inhibitor treatment promotes the phosphorylation and inactivation of GSK3β, thereby facilitating the nuclear translocation and activation of β-catenin. In contrast, AKT inhibitor treatment completely abrogates β-catenin activation and prevents GSK3β inactivation, which is consistent with the established role of AKT in mediating GSK3β phosphorylation and subsequent inactivation, thereby relieving the inhibitory effect on β-catenin. Furthermore, immunofluorescence analysis revealed that AKT1 protein expression is consistently localized to the nucleus, whereas the subcellular localization of the PI3K p85 regulatory subunit undergoes significant changes before and after differentiation. Collectively, these findings suggest that PI3K and AKT kinases play distinct roles during early mesoderm differentiation, with AKT kinase activity being indispensable for this process.

Indeed, previous studies have demonstrated that overexpression of Wnt ligands or Disheveled (Dvl) can activate AKT kinase [25]. In this study, we found that AKT phosphorylation is stimulated by Wnt signals and AKT inhibitors block the expression of mesodermal differentiation marker genes in the presence or absence of the Wnt agonist. Therefore, we concluded that during mesodermal differentiation, PI3K kinase activity is inhibited, while AKT kinase is activated via a Wnt-dependent mechanism.

Why does PI3K pathway inhibition promote mesoderm differentiation? Our transcriptomic sequencing data revealed that PI3K pathway inhibition activates the Wnt, Notch, and Nodal signaling cascades. Specifically, treatment with LY294002 upregulated the expression of *Wnt3*, *Wnt3a*, and *Wnt8*, as well as *Notch1*, *Dll1*, and *Dll3*, along with *Nodal* and *Tgfbr1*. Among these, Wnt3a is essential for cardiomyocyte differentiation, particularly for mesoderm specification [26]. Notably, in the cardiac differentiation model of ES cells, *Nodal* expression peaks within the first 1–2 days and then rapidly declines. In the absence of Nodal/Activin signaling, ES cells default to a neuroectodermal fate rather than the precursor cells required for cardiovascular development [10]. Our study revealed that the promoter of the mesoderm differentiation marker gene *Brachyury T* contains responsive elements for Wnt, Notch, and Nodal/Activin signaling pathways. Activation of Activin/Nodal signaling upregulates *Brachyury* T expression, whereas inhibition of either Wnt or Notch signaling suppresses its expression.

Previous studies have demonstrated that the PI3K-AKT signaling pathway plays a central and essential role in maintaining stem cell self-renewal, making it a critical hub for regulating stemness. Activation of the PI3K-AKT signaling pathway is sufficient to maintain an undifferentiated phenotype and pluripotency in mouse and primate (e.g., cynomolgus monkey) ES cells, even in the absence of leukemia inhibitory factor (LIF) [27]. Systematic analysis of the PI3K-AKT pathway further revealed that IRS1 and negative regulators of PIP3 serve as key regulatory nodes for maintaining pathway homeostasis in self-renewing human embryonic stem cells [28]. Our transcriptomic sequencing data revealed that PI3K pathway inhibition does not affect the expression of pluripotency genes such as *Oct4 (Pou5f1), Sox2, Nanog, and Klf4*. In fact, previous studies have reported that OCT4 and NANOG are essential for mesodermal and cardiac differentiation [29,30]. Activating the Wnt signaling pathway while simultaneously knocking out OCT4 leads to the inhibition of mesodermal gene expression [31]. Therefore, it is reasonable that PI3K inhibition does not affect the expression of genes such as *Oct4*, *Sox2*, *Nanog*, and *Klf4*.

Our data also indicated that PI3K inhibitors modulate the expression of multiple growth factors, their corresponding receptors, and intracellular adaptor proteins. The expression of *Tdgf1* and *Lif* was markedly decreased upon differentiation and further suppressed by PI3K inhibitor treatment. Notably, we found that *Grb7* and *Grb10* were downregulated during differentiation, and their expression was further reduced in the presence of PI3K inhibitors. The GRB7 (growth factor receptor-bound protein 7) adaptor protein family comprises three members: GRB7, GRB10, and GRB14. These adaptor proteins directly bind to phosphorylated receptor tyrosine kinase sites, including those of EGF and PDGF receptors as well as focal adhesion kinase complexes, through their SH2 domains, thereby regulating diverse cellular functions such as proliferation and migration. Previous studies have demonstrated that the p85 regulatory subunit of PI3K can form a complex with GRB7. Furthermore, GRB7 has been identified as a marker gene for undifferentiated pluripotent stem cells and is also implicated in the acquisition of stemness in cancer stem cells [32,33,34]. GRB10 has also been identified as a key factor associated with the pluripotent state of nuclear transfer ES cells [35]. The expression changes in growth factors, receptors, and intracellular adaptor proteins may be associated with the following two alterations: first, the downregulation of endogenous PI3K activity following cell differentiation. Second, upon cell differentiation, AKT is activated through a PI3K-independent pathway and plays a role in promoting differentiation. Overall, our study reveals a regulatory mechanism governing stem cell self-renewal and early differentiation: stemness-related genes such as OCT4 remain unchanged, whereas various growth factors and signaling molecules that regulate the PI3K-AKT pathway undergo significant alterations. The specific regulatory mechanisms require further in-depth investigation and elucidation.

Taken together, our findings suggest that the PI3K-AKT signaling pathway plays a regulatory role in stem cell self-renewal. Upon the initiation of cardiomyocyte differentiation, the PI3K pathway is suppressed, which in turn activates the Wnt, Notch, and Nodal signaling cascades, thereby triggering the mesoderm differentiation program. During the mesoderm differentiation stage, AKT kinase must be activated to inhibit GSK3β, consequently facilitating the activation of β-catenin. AKT activation during mesoderm differentiation occurs independently of the PI3K pathway but rather in a Wnt-dependent manner. Collectively, these findings provide important insights into the stage-specific roles of the PI3K-AKT pathway in cardiomyocyte differentiation.

## 5. Conclusions

PI3K inhibitors promote early myocardial differentiation and enhance the expression of the mesodermal differentiation marker gene *Brachyury T* via regulating Wnt/β-catenin, Notch, and Nodal signals. For mesodermal differentiation, AKT kinase activity is indispensable for GSK3β phosphorylation and β-catenin activation, and AKT inhibitors exert the inhibitory effects. AKT is activated during mesoderm differentiation in a Wnt-dependent manner.

## Figures and Tables

**Table 1:** Primers for qRT-PCR analysis.

Gene Name:	Primer Sequence:
*Brachyury T*	Fw: GCTTCAAGGAGCTAACTAACGAG
	Re: CCAGCAAGAAAGAGTACATGGC
*Nkx2.5*	Fw: CAAGTGCTCTCCTGCTTTCC
	Re: GGCTTTGTCCAGCTCCACT
*Myl7*	Fw: GGCACAACGTGGCTCTTCTAA
	Re: GATTTGCAGATGATCCCATCCC
*Wnt3a*	Fw: AATTTGGAGGAATGGTCTCTCGG
	Re: CAGCAGGTCTTCACTTCACAG
*Fgf8*	Fw: CCGAGGAGGGATCTAAGGAAC Re: CTTCCAAAAGTATCGGTCTCCAC
	Fw: GCGCATGTTTCCTTTCTTGAG Re: GGTCGGCCAGAACCACTTC
*Eomes*	Fw: TTCAAGCCTGTTGGGCTCTAC Re: TCCGGTCACGTCCACATCTT
	Fw: CAGGACCTTCTTTCGCGTATG Re: AAGGGGAATCGGATGGGGTT
*Nodal*	Fw: CCGAGGAGGGATCTAAGGAAC Re: CTTCCAAAAGTATCGGTCTCCAC
	Fw: GCGCATGTTTCCTTTCTTGAG Re: GGTCGGCCAGAACCACTTC
*Dll1*	Fw: TTCAAGCCTGTTGGGCTCTAC Re: TCCGGTCACGTCCACATCTT
	Fw: CAGGACCTTCTTTCGCGTATG Re: AAGGGGAATCGGATGGGGTT
*18s*	Fw: GTAACCCGTTGAACCCCAATT
	Re: CCATCCAATCGGTAGTAGCG

## Data Availability

The original contributions presented in this study are included in the article/Supplementary Materials. Further inquiries can be directed to the corresponding author.

## Supplementary Materials

The following supporting information can be downloaded at: https://www.mdpi.com/article/10.3390/biology15201887/s1, Original Western blot images for Figure 4 and Figure 8.
